# Development and Validation of a Predictive Model for Severe Tubular Atrophy/Interstitial Fibrosis in Patients with IgA Nephropathy: Multicenter Retrospective Study

**DOI:** 10.2196/78761

**Published:** 2025-10-28

**Authors:** Caizheng Yu, Zhitong Niu, Qin Fang, Qing Lei

**Affiliations:** 1 Department of Public Health, Tongji Hospital, Tongji Medical College, Huazhong University of Science and Technology, Wuhan, China; 2 Shanxi Bethune Hospital, Shanxi Academy of Medical Sciences, Third Hospital of Shanxi Medical University, Tongj Shanxi Hospital, Taiyuan, China; 3 Department of Medical Affairs, Zhuhai People's Hospital (The Affiliated Hospital of Beijing Institute of Technology, Zhuhai Clinical Medical College of Jinan University), Zhuhai, China; 4 Division of Nephrology, Tongji Hospital, Tongji Medical College, Huazhong University of Science and Technology, 1095 Jiefang Ave, Wuhan, 430030, China, 86 18086452883

**Keywords:** tubular atrophy/interstitial fibrosis, IgA nephropathy, predictive model, machine learning, internal and external validation

## Abstract

**Background:**

Severe tubular atrophy/interstitial fibrosis are critical pathological features associated with poor prognosis in IgA nephropathy (IgAN). The early identification of patients at high risk for severe tubular damage could guide clinical management and improve outcomes.

**Objective:**

This study aimed to construct and validate a predictive model for assessing the risk of severe tubular atrophy and interstitial fibrosis in patients diagnosed with IgAN.

**Methods:**

A total of 3276 patients from the Hankou branch of Tongji Hospital were retrospectively enrolled for model development. A predictive model for severe tubular atrophy/interstitial fibrosis was constructed based on independent predictors identified through univariate analysis, least absolute shrinkage and selection operator regression, and stepwise logistic regression. Furthermore, the model underwent internal and external validation using an independent dataset (n=1062), and performance evaluation using six machine learning algorithms: random forest, generalized linear model, decision tree, gradient boosting decision tree, extreme gradient boosting, and support vector machine.

**Results:**

In this study, 8 variables were identified as independent predictors and used to construct a predictive model for severe tubular atrophy/interstitial fibrosis: Logit (P)=0.011×age (years)+0.324×hypertension history–0.302×education+.111×coefficient of variation of red cell distribution width–0.152×direct bilirubin (μmol/L)+0.003×uric acid (μmol/L)–0.021×estimated glomerular filtration rate (ml/min/1.73m²)+1.151×ln(24 h urine microalbumin) (mg/24h). The AUC for the predictive model was 0.860 (95% CI 0.847‐0.873). The AUCs (95% CI) of the six machine learning algorithms ranged from 0.793 (0.765‐0.822) to 0.880 (0.859‐0.902) in internal validation and from 0.785 (0.756‐0.814) to 0.862 (0.839‐0.885) in external validation.

**Conclusions:**

We developed a concise and clinically useful model for predicting severe tubular atrophy/interstitial fibrosis in IgA nephropathy. It offers a non-invasive tool for risk assessment when biopsy is not feasible, aiding personalized treatment decisions.

## Introduction

Immunoglobulin A nephropathy (IgAN) is the most common form of primary glomerulonephritis worldwide, particularly in China [1,2]. As IgAN progresses, the glomerular filtration rate gradually declines, and approximately 30%‐40% of patients develop end-stage renal disease (ESRD) within 20 to 30 years after the onset of clinical symptoms, imposing a substantial burden on both individuals and health care systems [3,4].

The hallmark pathological feature of IgAN is the deposition of IgA in the glomerular mesangium, and renal biopsy remains the gold standard for diagnosis. According to the current Oxford classification guidelines [5], the pathological lesions in IgAN include mesangial hypercellularity (M), endocapillary hypercellularity (E), segmental glomerulosclerosis (S), tubular atrophy/interstitial fibrosis (T), and cellular or fibrocellular crescents (C), that is, MEST-C. Among these, tubular atrophy and interstitial fibrosis (T lesions) are considered key prognostic indicators [6]. The Oxford derivation and North American validation studies, along with the VALIGA study, with a median follow-up of 5.6 years, demonstrated that severe tubular atrophy and interstitial fibrosis were significantly associated with an increased risk of renal function decline or progression to ESRD [7]. Similarly, a multicenter retrospective cohort study from China involving 2047 patients with IgAN confirmed that severe tubular atrophy and interstitial fibrosis markedly elevated the risk of renal function deterioration or ESRD [8]. These findings indicated that severe tubular atrophy and interstitial fibrosis might lead to poor prognosis over long-term follow-up. However, renal biopsy is an invasive procedure that requires technical expertise, is costly, and is poorly accepted by patients, making it difficult to perform repeatedly. These limitations hinder its broad application in the long-term management of chronic kidney disease (CKD).

Predictive models are increasingly recognized as valuable tools for early risk stratification, particularly when renal biopsy is infeasible or repeat biopsy is not practical due to clinical or ethical concerns. Such models can facilitate precision medicine by informing treatment decisions, follow-up scheduling, and lifestyle modifications, thereby improving disease management while reducing unnecessary hospitalizations and health care expenditures. However, existing predictive models [8-10] for tubular atrophy and interstitial fibrosis in IgAN are often limited by small sample sizes, lack of validation, and limited feasibility in clinical practice. Therefore, developing a simple, practical, and reliable model to predict tubular atrophy and interstitial fibrosis in patients with IgAN is of great clinical and social importance.

In this study, we aimed to construct a predictive model for tubular atrophy and interstitial fibrosis in IgAN by integrating multidimensional data from hospitalized patients who underwent renal biopsy at the Department of Nephrology, Tongji Hospital. The model was developed using least absolute shrinkage and selection operator (LASSO) regression and logistic regression, and was internally and externally validated using six different machine learning algorithms.

## Methods

### Study Population

Participants were recruited from three medical centers located in Wuhan, China, including Tongji Hospital Hankou branch (Qiaokou District), the Tongji Hospital Optics Valley branch (Donghu High-Tech Development Zone), and the Tongji Hospital Sino-French New City branch (Caidian District). Demographic characteristics, lifestyle factors, medical history, and laboratory test results were extracted from the electronic medical record system. Specifically, we retrospectively enrolled 3970 patients who were hospitalized in the Department of Nephrology at the Tongji Hospital Hankou branch between 2004 and 2023 and who underwent renal biopsy during hospitalization. The following exclusion criteria were applied: (1) age ≤18 years (n=98); (2) number of glomeruli <8 in the biopsy specimen (n=32); (3) history of tumor or transplantation (n=103); (4) variables with more than 20% missing values were excluded, that is, 66 variables were included; participants with more than 20% missing data among these 66 variables were further excluded (n=461). Finally, 3276 patients were included as the population for model development. The study design flowchart is presented in Figure S1 in Multimedia Appendix 1. An independent validation sample consisting of 1062 patients was enrolled from the Nephrology Divisions of Tongji Hospital’s Sino-French New City and Optics Valley branches, who underwent renal biopsy between 2015 and 2023. Detailed exclusion criteria for the validation population are provided in Figure S2 in Multimedia Appendix 1.

### Data Collection

With the support of the Big Data Center of Tongji Hospital, data on demographic characteristics (age, sex, education level, height, and weight), lifestyle factors (smoking and alcohol consumption), medical history (hypertension and diabetes), and laboratory test results (blood and urine tests) were extracted from the electronic medical record system. Educational attainment was categorized as “middle school or below” and “high school or above.” The BMI was calculated as weight in kilograms divided by height in meters squared. Within 24 hours of hospital admission, fresh whole blood samples were collected from each patient for routine blood tests and assessments of liver function, renal function, lipid profile, and fasting blood glucose levels. In addition, morning urine and 24-hour urine samples were collected during hospitalization for routine urinalysis and 24-hour urine biochemical tests, including urinary protein and renal function markers. All blood and urine examinations were conducted by trained technicians in the clinical laboratory of Tongji Hospital, following uniform testing protocols. To reduce skewness, natural logarithmic transformation was applied to the following variables: urinary creatinine, urine albumin-to-creatinine ratio, urinary micro total protein, urinary microalbumin, 24-hour urine total microprotein, and 24-hour urine microalbumin.

### Renal Biopsy Evaluation

All study participants underwent renal biopsy. Each biopsy specimen was subjected to light microscopy, immunofluorescence staining, and electron microscopy examination. For light microscopy analysis, slides were stained with hematoxylin and eosin, periodic acid–Schiff, Masson’s trichrome, and silver staining, following standardized protocols. All renal pathology assessments were performed by experienced renal pathologists and classified according to the Oxford MEST-C scoring system for IgAN. Light microscopic findings of IgA nephropathy range from mild mesangial hypercellularity to diffuse mesangial proliferation, which may be accompanied by segmental sclerosis, glomerular adhesions, and/or crescent formation. Immunofluorescence predominantly showed mesangial staining of IgA. Electron microscopy revealed mesangial deposits along with mesangial matrix expansion (Figure S3 in Multimedia Appendix 1). In the MEST-C score, the T component refers to tubular atrophy and interstitial fibrosis: T0 corresponds to ≤25%, T1 to 26%‐50%, and T2 to >50%. In this study, renal biopsy specimens classified as T0 were defined as mild tubular atrophy/interstitial fibrosis, whereas T1 and T2 were grouped as severe tubular atrophy/interstitial fibrosis (Figure S4 in Multimedia Appendix 1).

### Statistical Analysis

Data are presented as median (Q1–Q3) for continuous variables and number (%) for categorical variables. The Shapiro-Wilk test was used to assess the normality of all continuous variables. For variables that followed a normal distribution, between-group differences were assessed using the independent samples *t* test; for non-normally distributed variables, the Mann-Whitney *U* test was employed. Variables showing significant between-group differences were further analyzed using least absolute shrinkage and selection operator (LASSO) regression to identify predictors of severe tubular atrophy/interstitial fibrosis. To address multicollinearity and enhance model stability, variables with a variance inflation factor greater than 5 were excluded. Subsequently, stepwise backward logistic regression was performed on the LASSO-selected candidate predictors to determine independent influencing factor for the severity of tubular atrophy/interstitial fibrosis. Model parameters were estimated, and the prediction model was constructed as follows: Logit (P=*β*0+Variable1 × *β*1+Variable2 × *β*2 +…+VariableX×*β*X). The effects of the predictors on tubular atrophy/interstitial fibrosis severity were visualized using a forest plot. A nomogram was developed to provide a graphical representation of the IgAN tubular atrophy/interstitial fibrosis prediction model. The predictive performance and calibration of the model were evaluated by receiver operating characteristic (ROC) curves, calibration curves, and decision curve analysis (DCA). To evaluate the overall accuracy of the predictive model, we calculated the Brier score, which measures the mean squared difference between the predicted probabilities and the observed outcomes. In addition, the Youden index was used to determine the optimal cutoff value for classification by maximizing the sum of sensitivity and specificity. For internal validation, the development population was randomly split into training and testing sets at a ratio of 7:3. An external validation cohort consisting of 1062 patients hospitalized at the Sino-French New City and Optics Valley branches of Tongji Hospital between 2015 and 2023, who underwent renal biopsy, was used to validate the model’s performance.

During both internal and external validations, five-fold cross-validation was conducted combined with six machine learning algorithms—random forest, generalized linear model, decision tree, gradient boosting decision tree, extreme gradient boosting, and support vector machine. Model performance and discriminative ability were assessed using accuracy, precision, recall, F_1_-score, area under the ROC curve (AUC), and precision-recall curves. All statistical analyses were performed using R software (version 4.4.1; R Foundation for Statistical Computing). A two-sided *P* value <.05 was considered statistically significant.

### Ethical Considerations

This study was approved by the Ethics Committee of Tongji Hospital (approval number: TJ-IRB202410023), and written informed consent was obtained from all participants. In addition, the patient information included in this study has been deidentified. No compensation was provided to any participants.

## Results

A total of 3276 hospitalized patients with biopsy-confirmed IgA nephropathy were included in this study. The characteristics of individuals with mild and severe tubular atrophy/interstitial fibrosis are presented in Table 1. Among the 3276 participants, 2086 (63.7%) had mild tubular atrophy/interstitial fibrosis, whereas 1190 (36.3%) had severe tubular atrophy/interstitial fibrosis. Compared to those with mild tubular atrophy/interstitial fibrosis, individuals with severe tubular atrophy/interstitial fibrosis were older, more likely to be male, had lower educational attainment, and exhibited higher proportions of hypertension, diabetes, smoking, and alcohol consumption, as well as higher BMI (all *P*<.05). In addition, significant differences were observed between the two groups in terms of hematological parameters, liver function, renal function, lipid profiles, and glucose metabolism indicators (all *P*<.05).

**Table 1.** Characteristics of study individuals and univariates analysis of mild/severe tubular atrophy/interstitial fibrosis groups (n=3276).

Variables	Mild group (n=2086)	Severe group (n=1190)	*P* value
Age, years, median (IQR)	34.0 (27.0‐43.0)	36.0 (29.0‐46.0)	<.001
Women, n (%)	1198 (57.4)	592 (49.8)	<.001
Hypertension history, n (%)	558 (26.8)	557 (46.8)	<.001
Diabetes history, n (%)	195 (9.4)	179 (15.0)	<.001
Smoking, n (%)	164 (7.9)	152 (12.8)	<.001
Alcohol consumption, n (%)	116 (5.6)	96 (8.1)	.006
Education (high school or above), n (%)	1420 (68.1)	700 (58.8)	<.001
Systolic blood pressure, mmHg, median (IQR)	124.0 (113.0‐135.0)	132.0 (120.0‐144.0)	<.001
Diastolic blood pressure, mmHg, median (IQR)	82.0 (75.0‐90.0)	86.0 (78.0‐96.0)	<.001
BMI, kg/m², median (IQR)	22.7 (20.4‐24.9)	23.1 (20.8‐26.0)	<.001
White blood cell count, ×10⁹/L, median (IQR)	6.7 (5.7‐8.0)	7.0 (5.9‐8.5)	<.001
Neutrophil percentage, median (IQR)	63.2 (57.4‐68.8)	64.9 (59.2‐70.5)	<.001
Neutrophil count, ×10⁹/L, median (IQR)	4.2 (3.3‐5.3)	4.5 (3.6‐5.7)	<.001
Lymphocyte percentage, median (IQR)	27.8 (22.5‐33.1)	26.1 (20.9‐30.9)	<.001
Lymphocyte count, ×10⁹/L, median (IQR)	1.9 (1.5‐2.2)	1.8 (1.4‐2.2)	.003
Monocyte percentage, median (IQR)	6.5 (5.4‐7.8)	6.4 (5.1‐7.8)	.017
Monocyte count, ×10⁹/L, median (IQR)	0.4 (0.4‐0.6)	0.4 (0.4‐0.6)	.122
Eosinophil percentage, median (IQR)	1.5 (0.9‐2.4)	1.6 (0.9‐2.7)	.030
Eosinophil count, ×10⁹/L, median (IQR)	0.1 (0.1‐0.2)	0.1 (0.1‐0.2)	.018
Basophil percentage, median (IQR)	0.3 (0.2‐0.5)	0.3 (0.2‐0.5)	.421
Basophil count, ×10⁹/L, median (IQR)	0.0 (0.0‐0.0)	0.0 (0.0‐0.0)	.064
Red blood cell count, ×10¹²/L, median (IQR)	4.4 (4.0‐4.9)	4.3 (3.8‐4.7)	<.001
Hemoglobin, g/L, median (IQR)	132.0 (120.0‐145.0)	125.0 (111.0‐140.0)	<.001
Hematocrit percentage, median (IQR)	39.1 (35.8‐42.6)	37.2 (33.4‐41.4)	<.001
MCV^a^, fL, median (IQR)	88.3 (85.6‐91.1)	88.2 (85.2‐91.1)	.223
MCH^b^, pg, median (IQR)	29.9 (28.7‐30.9)	29.9 (28.7‐30.8)	.931
MCHC^c^, g/L, median (IQR)	337.0 (328.0‐345.0)	337.0 (328.0‐346.0)	.583
RDW-CV^d^, median (IQR)	12.6 (12.1‐13.2)	12.8 (12.2‐13.5)	<.001
RDW-SD^e^, fL, median (IQR)	40.5 (38.7‐42.6)	40.8 (39.0‐43.1)	<.001
Platelet count, ×10⁹/L, median (IQR)	226.0 (187.3‐271.0)	229.0 (185.0‐271.0)	.552
Platelet distribution width, fL, median (IQR)	12.8 (11.4‐14.8)	12.8 (11.4‐14.7)	.900
Mean platelet volume, fL, median (IQR)	10.6 (9.9‐11.4)	10.7 (10.0‐11.5)	.538
Large platelet ratio, %, median (IQR)	30.7 (25.3‐37.6)	31.1 (25.3‐37.4)	.911
Plateletcrit, %, median (IQR)	0.2 (0.2‐0.3)	0.2 (0.2‐0.3)	.905
ALT^f^, U/L, median (IQR)	14.0 (11.0‐22.0)	14.0 (10.0‐21.0)	.361
AST^g^, U/L, median (IQR)	18.0 (15.0‐21.0)	18.0 (15.0‐22.0)	.602
Total protein, g/L, median (IQR)	71.7 (67.4‐75.6)	68.8 (63.2‐73.0)	<.001
Albumin, g/L, median (IQR)	42.2 (38.9‐45.2)	39.3 (35.3‐42.8)	<.001
Globulin, g/L, median (IQR)	29.2 (26.9‐31.8)	28.9 (26.3‐32.0)	.077
Total bilirubin, μmol/L, median (IQR)	7.7 (5.5‐10.3)	6.6 (4.5‐9.3)	<.001
Direct bilirubin, μmol/L, median (IQR)	2.4 (1.9‐3.3)	2.1 (1.6‐2.9)	<.001
Indirect bilirubin, μmol/L, median (IQR)	5.4 (3.6‐7.5)	4.9 (3.1‐7.1)	<.001
ALP^h^, U/L, median (IQR)	60.0 (50.0‐74.0)	61.0 (50.0‐75.0)	.692
GGT^i^, U/L, median (IQR)	17.0 (13.0‐25.0)	19.0 (13.3‐28.0)	<.001
Total cholesterol, mmol/L, median (IQR)	4.4 (3.8‐5.0)	4.6 (3.9‐5.5)	<.001
Triglycerides, mmol/L, median (IQR)	1.4 (1.0‐2.1)	1.8 (1.2‐2.6)	<.001
High-density lipoprotein, mmol/L, median (IQR)	1.1 (1.0‐1.4)	1.1 (0.9‐1.3)	<.001
Low-density lipoprotein, mmol/L, median (IQR)	2.6 (2.1‐3.1)	2.8 (2.2‐3.5)	<.001
Urea, mmol/L, median (IQR)	5.1 (4.1‐6.2)	6.8 (5.2‐9.1)	<.001
Serum creatinine, μmol/L, median (IQR)	80.0 (64.0‐100.0)	116.5 (84.0‐165.0)	<.001
Uric acid, μmol/L, median (IQR)	333.0 (272.9‐402.0)	394.0 (328.8‐473.9)	<.001
eGFR^j^, ml/min/1.73 m², median (IQR)	96.5 (75.5‐115.2)	61.4 (40.0‐90.5)	<.001
Erythrocyte sedimentation rate, mm/h, median (IQR)	8.0 (4.0‐14.0)	13.0 (6.0‐26.0)	<.001
Fasting blood glucose, mmol/L, median (IQR)	5.3 (4.9‐6.0)	5.4 (4.9‐6.3)	<.001
ALT/AST ratio, median (IQR)	0.8 (0.7‐1.1)	0.8 (0.6‐1.1)	.256
Albumin/Globulin ratio, median (IQR)	1.4 (1.3‐1.6)	1.3 (1.2‐1.5)	<.001
ln(Urinary creatinine), μmol/L, median (IQR)	9.5 (9.0‐9.9)	9.2 (8.8‐9.6)	<.001
ln(Urine albumin-to-creatinine ratio), μg/mg, median (IQR)	5.8 (5.0‐6.6)	6.9 (6.1‐7.7)	<.001
ln(Urinary micro total protein), mg/L, median (IQR)	6.4 (5.8‐7.1)	7.3 (6.6‐7.9)	<.001
ln(Urinary microalbumin), mg/L, median (IQR)	6.1 (5.3‐6.8)	7.0 (6.3‐7.6)	<.001
ln(24 h urine total microprotein), mg/24 h, median (IQR)	6.4 (5.8‐6.9)	7.4 (7.0‐7.9)	<.001
ln(24 h urine microalbumin), mg/24 h, median (IQR)	6.0 (5.3‐6.6)	7.2 (6.7‐7.7)	<.001
Urine pH, median (IQR)	6.0 (5.5‐6.5)	6.0 (5.5‐6.5)	.060
Urine specific gravity, median (IQR)	1.0 (1.0‐1.0)	1.0 (1.0‐1.0)	.003
Urinary red blood cell count/μL, median (IQR)	35.7 (12.2‐112.4)	28.1 (9.7‐76.4)	<.001
Urinary white blood cell count/μL, median (IQR)	7.8 (3.6‐19.5)	7.3 (3.4‐17.0)	.065

a MCV: Mean corpuscular volume.

b MCH: Mean corpuscular hemoglobin.

c MCHC: Mean corpuscular hemoglobin concentration.

d RDW-CV: coefficient of variation of red blood cell volume size.

e RDW-SD: standard deviation of red blood cell distribution width.

f ALT: Alanine aminotransferase.

g AST: Aspartate aminotransferase.

h ALP: Alkaline phosphatase.

i GGT: Gamma-glutamyl transferase.

j eGFR: Estimated glomerular filtration rate.

**Figure 1.**
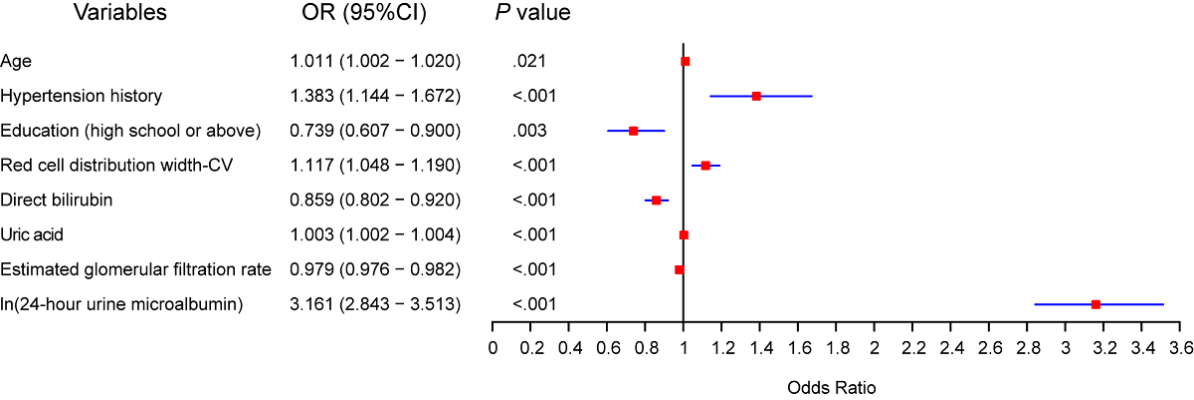
Forest plot of independent predictors for severe tubular atrophy/interstitial fibrosis identified on logistic regression analysis.

Univariate analysis revealed that 48 out of 66 analyzed variables showed statistically significant differences between the mild and severe tubular atrophy/interstitial fibrosis groups. These 48 variables were subsequently included in a LASSO regression model to reduce dimensionality and identify potential predictors. The LASSO analysis yielded 35 candidate predictors (Figure S5 in Multimedia Appendix 1). To further reduce multicollinearity and enhance model stability, variables with a variance inflation factor greater than 5 were excluded, resulting in 31 remaining candidate predictors (Figure S6 in Multimedia Appendix 1). Furthermore, these 31 predictors were then subjected to stepwise backward logistic regression analysis. Eight variables were ultimately identified as independent predictors of severe tubular atrophy/interstitial fibrosis: age (OR 1.011; 95% CI 1.002‐1.020; *P*=.021), hypertension history (OR 1.383; 95% CI 1.144‐1.672; *P*<.001), education (high school or above; OR 0.739; 95% CI 0.607‐0.900; *P*=.003), coefficient of variation of red blood cell volume size (red cell distribution width-CV; OR 1.117; 95% CI 1.048‐1.190; *P*<.001), direct bilirubin (OR 0.859; 95% CI 0.802‐0.920; *P*<.001), uric acid (OR 1.003; 95% CI 1.002‐1.004; *P*<.001), estimated glomerular filtration rate (eGFR; OR 0.979; 95% CI 0.976‐0.982; *P*<.001), and ln(24-hour urine microalbumin) (OR 3.161; 95% CI 2.843‐3.513; *P*<.001; Figure 1).

**Figure 2.**
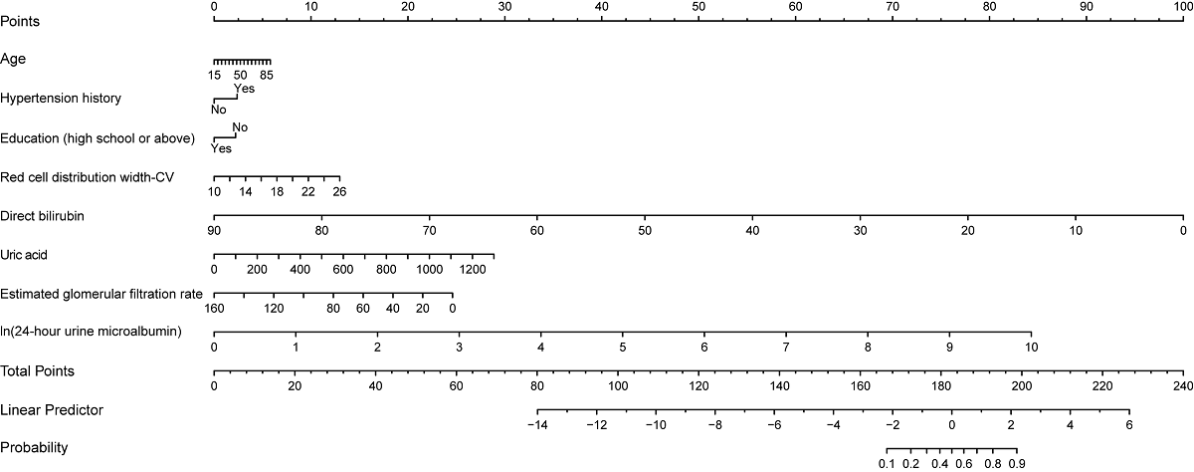
Nomogram for predicting the risk of severe tubular atrophy/interstitial fibrosis.

Based on these eight independent predictors, a logistic regression model was constructed to predict severe tubular atrophy/interstitial fibrosis. The final model equation was as follows: Logit (P)=0.011×age (years)+0.324×hypertension history –0.302×education+.111×red cell distribution width-CV –0.152×direct bilirubin (μmol/L)+0.003×uric acid (μmol/L) –0.021×eGFR (ml/min/1.73m²)+1.151×ln(24 h urine microalbumin) (mg/24h). In this equation, hypertension history was coded as 0=no, 1=yes; education level was coded as 0=junior high school or below, 1=high school or above. The predicted probability *P* ranges between 0 and 1. To facilitate clinical application, a nomogram was developed based on the final model (Figure 2). The nomogram included a point scale (0‐100 points), the eight independent predictors, a total score axis (0‐240 points), a linear predictor axis, and the predicted probability of severe tubular atrophy/interstitial fibrosis. This provided an intuitive and user-friendly visualization of the contribution of each predictor to the risk of severe tubular atrophy/interstitial fibrosis.

**Figure 3.**
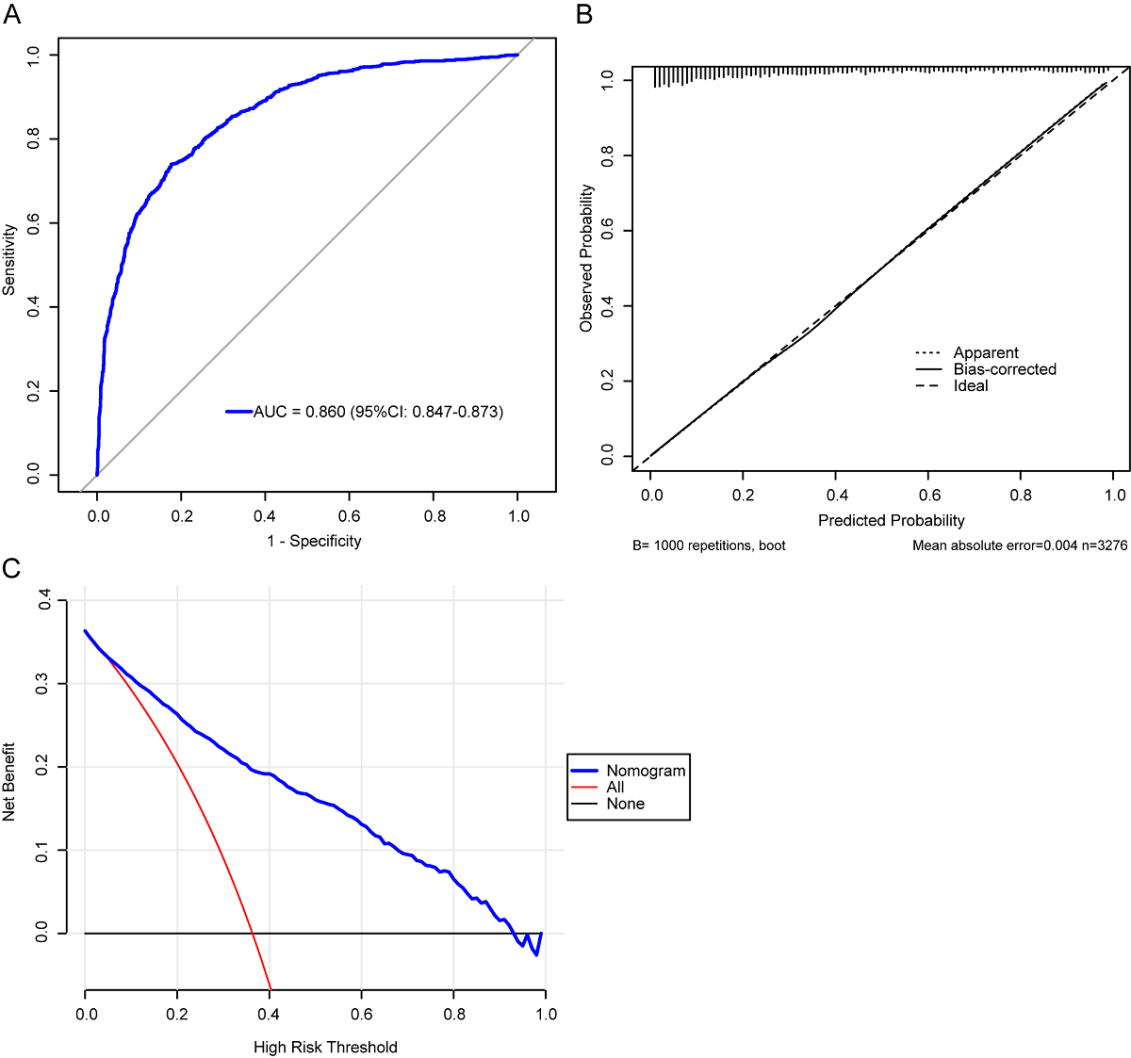
Performance evaluation of the predictive model for severe tubular atrophy/interstitial fibrosis. (A) Receiver operating characteristic (ROC) curve assessing the discrimination ability of the model. (B) Calibration curve evaluating the agreement between predicted and observed outcomes. (C) Decision curve analysis (DCA) demonstrating the net clinical benefit across different threshold probabilities.

**Figure 4.**
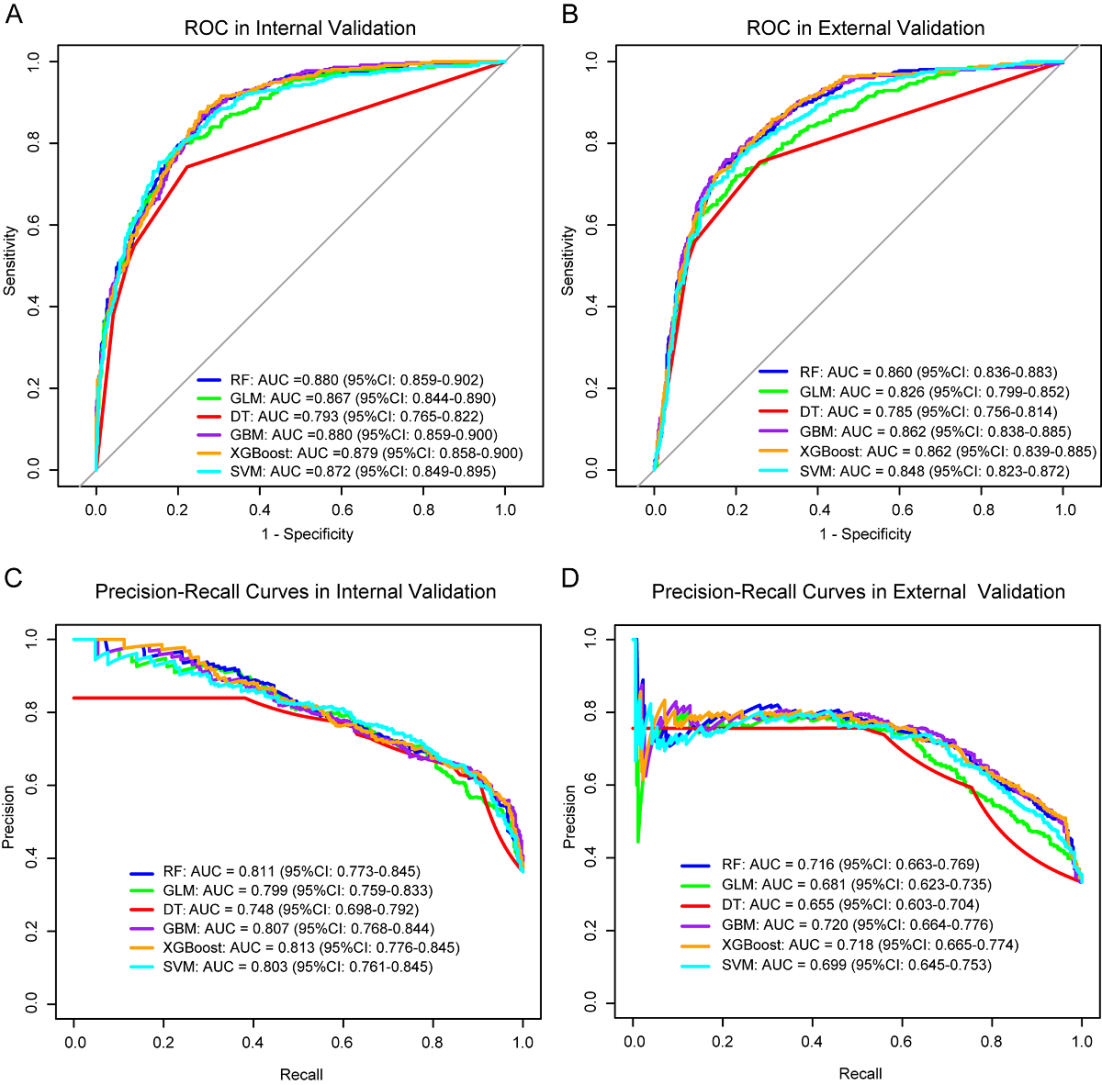
Internal and external validation of severe tubular atrophy/interstitial fibrosis using six machine learning algorithms. (A) Receiver operating characteristic (ROC) curves in internal validation. (B) ROC curves in external validation. (C) Precision-recall curves in internal validation. (D) Precision-recall curves in external validation. RF: random forest; GLM: generalized linear model; DT: decision tree, GBM: gradient boosting decision tree; XGBoost: extreme gradient boosting; SVM: support vector machine.

Model performance was evaluated using ROC, calibration plots, and DCA. The AUC for the logistic model was 0.860 (Figure 3A), indicating excellent discriminative ability. The calibration curve closely followed the diagonal line (y=x), with a mean absolute error of 0.004, suggesting a high agreement between predicted and observed risks (Figure 3B). DCA demonstrated favorable net benefits across a range of threshold probabilities from 0 to 1 (Figure 3C). The optimal cutoff value determined by the Youden index was 0.407, yielding a sensitivity of 73.9% and a specificity of 82.4%. Accordingly, patients with predicted probabilities ≥0.407 were classified as high risk for severe tubular atrophy and interstitial fibrosis. The Brier score was 0.143, indicating good calibration and probability prediction performance of the model. Internal and external validation of the prediction model was further performed using six machine learning algorithms. In internal validation, the AUCs (95% CI) of the ROC curves ranged from 0.793 (0.765‐0.822) to 0.880 (0.859‐0.902), while those of the precision-recall curves ranged from 0.748 (0.698‐0.792) to 0.813 (0.776‐0.845; Figure 4A and C). In external validation, ROC AUCs (95% CI) ranged from 0.785 (0.756‐0.814) to 0.862 (0.839‐0.885) and precision-recall AUCs from 0.655 (0.603‐0.704) to 0.720 (0.664‐0.776; Figure 4B and D). As summarized in Table 2, the logistic regression model demonstrated robust classification performance, with consistently high accuracy, precision, F_1_-score, and recall across both internal and external validation datasets. All six machine learning models exhibited comparable performances. During internal validation, accuracy ranged from 0.758 to 0.783, with recall values consistently high (0.742‐0.896). The model with the highest accuracy (0.783) also achieved the best F_1_-score (0.747) and recall (0.896). In external validation, the model accuracy ranged from 0.721 to 0.757, and recall remained satisfactory (0.754‐0.870). The top-performing model yielded an F_1_-score of 0.698 and a recall of 0.862, indicating good generalizability. Despite minor variations across models, precision was generally lower than recall in both validation sets, suggesting a trade-off between false positives and sensitivity.

**Table 2.** Performance metrics of six machine learning models in internal and external validation.

Models	Accuracy	Precision	F_1_-score	Recall
Internal validation				
RF^a^	0.775	0.635	0.743	0.896
GLM^b^	0.758	0.624	0.716	0.840
DT^c^	0.765	0.656	0.696	0.742
GBM^d^	0.778	0.643	0.742	0.877
XGBoost^e^	0.783	0.648	0.747	0.882
SVM^f^	0.777	0.654	0.728	0.821
External validation				
RF	0.748	0.581	0.697	0.870
GLM	0.721	0.558	0.654	0.791
DT	0.746	0.593	0.664	0.754
GBM	0.750	0.586	0.696	0.856
XGBoost	0.751	0.587	0.698	0.862
SVM	0.757	0.600	0.691	0.814

a RF: random forest.

b GLM: generalized linear model.

c DT: decision tree.

d GBM: gradient boosting decision tree.

e XGBoost: extreme gradient boosting.

f SVM: support vector machine.

## Discussion

### Principal Findings and Comparison With Previous Work

This study developed and validated a predictive model for severe tubular atrophy/interstitial fibrosis in hospitalized patients with IgA nephropathy at Tongji Hospital, based on multidimensional clinical data. The model was successfully constructed using 8 independent predictors significantly associated with severe tubular atrophy/interstitial fibrosis: age, hypertension history, education, red cell distribution width-CV, direct bilirubin, uric acid, eGFR, and ln(24-hour urine microalbumin). This model holds substantial clinical value in early diagnosis and screening, clinical decision-making, personalized treatment planning, and pathological monitoring in renal transplant recipients.

In line with previous studies, age and hypertension were identified as independent risk factors for severe tubular atrophy/interstitial fibrosis, highlighting their pivotal roles in CKD progression [11-14]. Advancing age is typically associated with a decline in nephron number, increased glomerulosclerosis, and exacerbated interstitial fibrosis [11,12]. Moreover, age-related inflammatory processes may contribute to irreversible structural kidney damage. Hypertension, a major driver of CKD progression, may promote ischemic injury in renal microvasculature and activate pro-fibrotic pathways, thereby accelerating tubular atrophy/interstitial fibrosis [13,15]. Interestingly, a higher education level (high school or above) was inversely associated with severe tubular atrophy/interstitial fibrosis. Previous cohort and Mendelian randomization studies have demonstrated a causal relationship between lower educational attainment and both increased risk of chronic kidney disease and reduced eGFR [16-18]. These data indicated that higher educational attainment might indeed play a role in preserving kidney function, including structural injury such as tubular atrophy and interstitial fibrosis. It was unlikely that education level directly influences tubular damage or fibrosis. Rather, it might act as a proxy for a range of interconnected downstream factors. Individuals with a higher education might have better health literacy, medication adherence, and proactive engagement with health care systems, enabling the earlier identification and management of modifiable risk factors like hypertension, diabetes, and metabolic syndrome [19]. Furthermore, prior studies have suggested that a low educational attainment might amplify the genetic risk for reduced eGFR [17], offering a potential mechanistic link from a genetic–environmental interaction perspective. Together, these findings support the plausibility that the education level is indirectly associated with tubular atrophy/interstitial fibrosis severity through both environmental and genetic pathways.

In this study, we observed that higher levels of RDW-CV were positively associated with tubular atrophy and interstitial fibrosis. RDW-CV, an index reported in the routine complete blood count test, reflects the variation in red blood cell size and is an established marker of erythrocyte volume heterogeneity. It has been linked to systemic inflammation and oxidative stress in various chronic diseases [20]. A large population-based cohort study revealed a strong graded association between RDW and C-reactive protein, which remained significant even after adjusting for multiple confounding factors [21]. Chronic inflammation and subsequent oxidative stress are thought to be key mediators linking elevated RDW to kidney injury [22]. In renal tissue, persistent inflammation and oxidative stress can activate NF-κB pathways, promoting fibroblast proliferation and extracellular matrix deposition [23,24]. Direct bilirubin, an endogenous antioxidant, has recently gained attention for its anti-inflammatory and anti-fibrotic properties. Mildly elevated bilirubin levels might confer protective effects against several chronic conditions, including diabetic nephropathy, metabolic syndrome, cardiovascular disease, and cancer [25-28]. For instance, a large cohort study demonstrated an inverse association between plasma bilirubin levels and all-cause mortality [29]. Mechanistically, bilirubin can modulate immune responses by influencing the expression of cell adhesion molecules and complement activity, as well as by inhibiting T cell differentiation [30]. It also suppresses the release of cytokines such as IL-2, IL-6, IL-10, and tumor necrosis factor-α, and reduces the expression of major histocompatibility complex class II in macrophages [25], thereby contributing to its immunomodulatory and anti-inflammatory effects. Additionally, bilirubin could alleviate renal inflammation and fibrosis through suppression of oxidative stress, inhibition of NADPH oxidase, and blockade of the TGF-β signaling pathway [25,31]. The inverse association observed in this study supports its potential nephroprotective role.

Elevated serum uric acid levels were known to exert pro-inflammatory effects and can induce tubular epithelial cell apoptosis and epithelial-to-mesenchymal transition [32,33]. In this study, we observed a positive association between serum uric acid levels and the severity of tubular atrophy and interstitial fibrosis. Consistent with our findings, previous studies have also reported that elevated serum uric acid contributes to the progression of renal dysfunction [34]. Several potential mechanisms might underlie this association. For instance, the accumulation of uric acid has been reported to induce mast cell degranulation and stimulate renin secretion, which subsequently enhances angiotensin II production and contributes to oxidative stress within renal tissue [35]. Animal studies have demonstrated that uric acid promotes interstitial damage and fibrosis through the activation of the NLRP3 inflammasome and ROS signaling pathways [36,37]. Clinically, hyperuricemia has been consistently associated with poor outcomes in IgAN [38]. Furthermore, both eGFR and 24-hour urinary microalbumin are established biomarkers reflecting glomerular and tubular damage [39,40]. Persistent microalbuminuria might indicate impaired tubular reabsorption and increased local inflammation. The predictive value of these markers for tubular atrophy/interstitial fibrosis was further corroborated in this study.

### Limitations

Although the study benefits from a relatively large sample size, strong statistical foundations, and clinical relevance, several limitations should be acknowledged. First, data were derived from three medical centers affiliated with Tongji Hospital in Wuhan, and although both internal and external validations were conducted, further validation in multi-center cohorts is necessary to assess the model’s generalizability. Second, the cross-sectional nature of the biopsy-based design limits the ability to predict the progression of tubular atrophy/interstitial fibrosis over time. Third, although our model included a comprehensive set of routinely available clinical and laboratory variables, key factors such as diabetes subtype, medication use (such as renin-angiotensin system inhibitors or immunosuppressants), and kidney imaging data were not captured in our dataset. The absence of these variables might have limited the model’s predictive accuracy and clinical interpretability. Future studies integrating multicenter cohorts, longitudinal follow-up, and additional biomarkers, medication history, or imaging features are needed to improve the model’s external validity and clinical applicability.

### Conclusions

In summary, this study developed a concise, accurate, and clinically interpretable predictive model for severe tubular atrophy/interstitial fibrosis in patients with IgA nephropathy, integrating multi-center data and machine learning techniques. This tool offers a non-invasive alternative for risk stratification of patients in whom renal biopsy is contraindicated or impractical, providing valuable support for clinical decision-making and promoting individualized management strategies.

## Supplementary material

10.2196/78761 Multimedia Appendix 1 Study and external validation flowcharts and supplementary materials regarding histopathological findings, least absolute shrinkage and selection operator regression findings, and variable inflation factor rankings.

## Abbreviations

AUC: area under the receiver operating characteristic curve
CKD: chronic kidney disease
DCA: decision curve analysis
eGFR: estimated glomerular filtration rate
ESRD: end-stage renal disease
IgAN: immunoglobulin A nephropathy
LASSO: least absolute shrinkage and selection operator
MEST-C: hypercellularity, endocapillary hypercellularity, segmental glomerulosclerosis, tubular atrophy/interstitial fibrosis, and cellular or fibrocellular crescents
ROC: receiver operating characteristic
