# Supplementary material for: Development and Validation of a Predictive Model for Severe Tubular Atrophy/Interstitial Fibrosis in Patients with IgA Nephropathy: Multicenter Retrospective Study
Source: JMIR Med Inform. 2025 Oct 28;13:e78761. doi: 10.2196/78761 (PMC12560959; doi:10.2196/78761)
Supplement: Multimedia Appendix 1 [file medinform-v13-e78761-s001.docx]

**
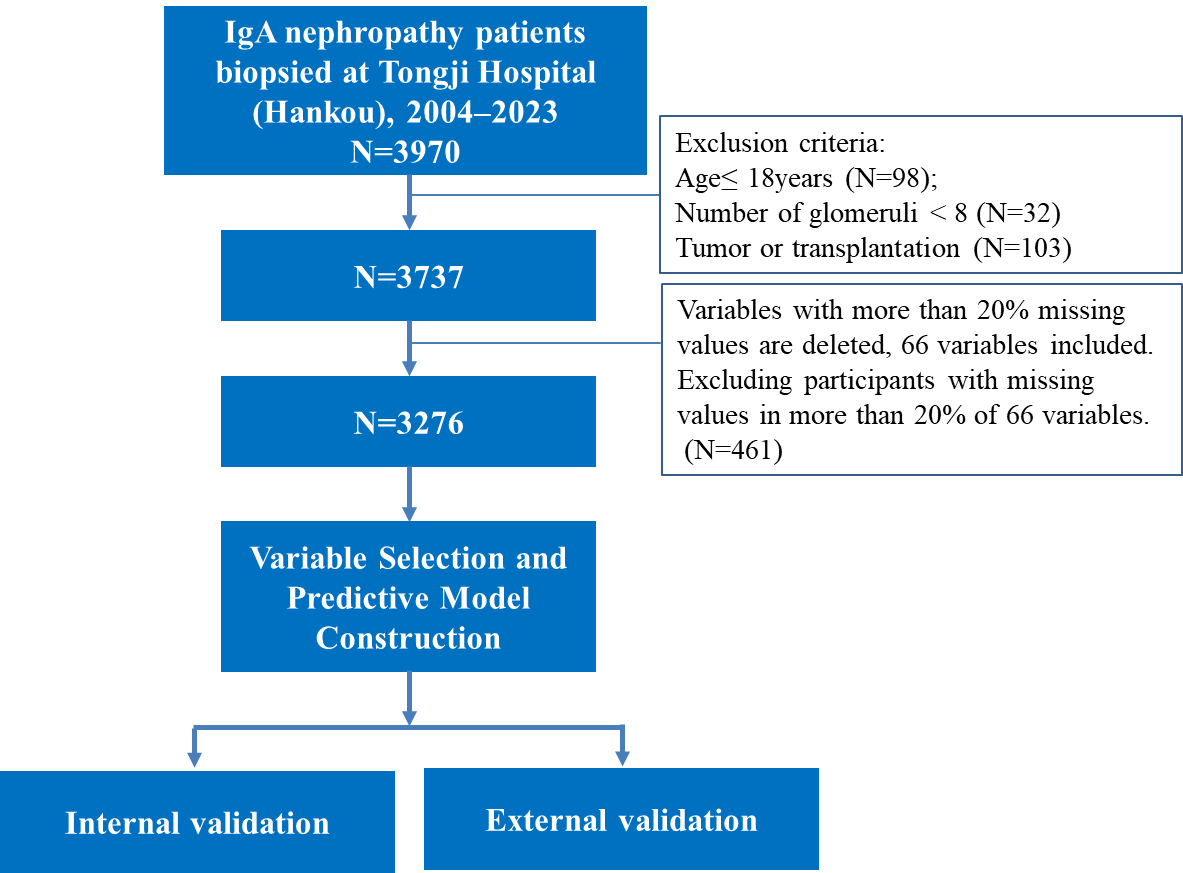
**

**Figure S1. Flowchart of the present study design.**


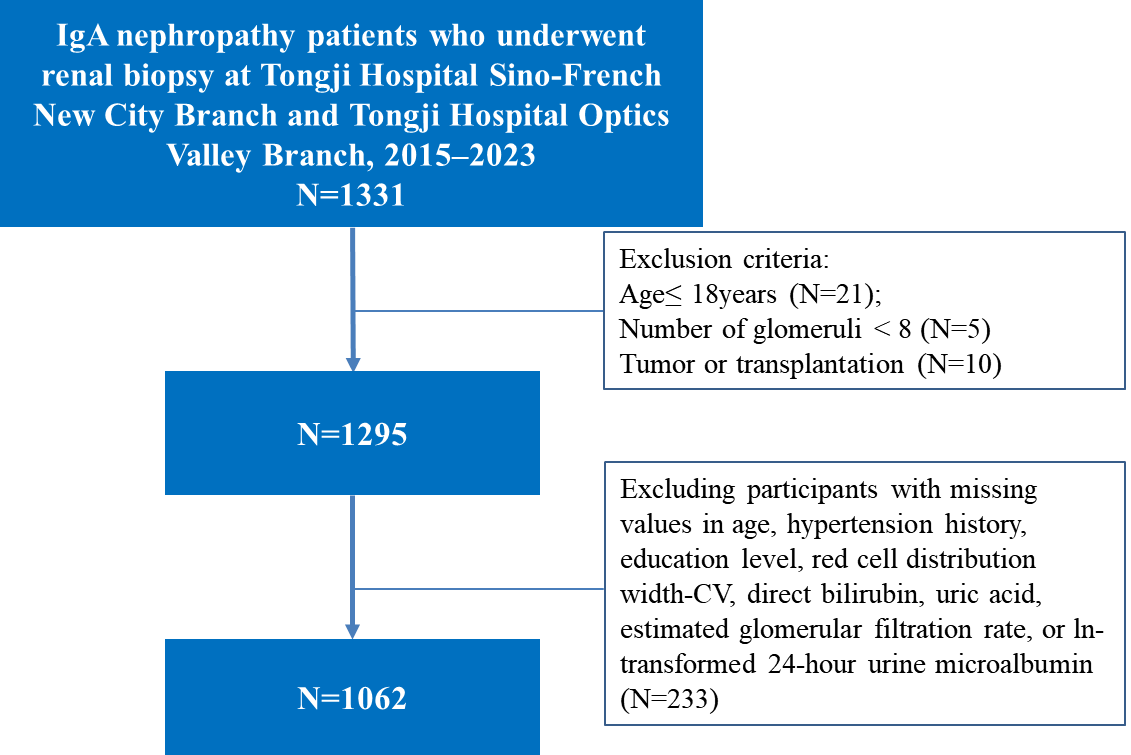


**Figure S2. Flowchart of the selection process for the external validation.**


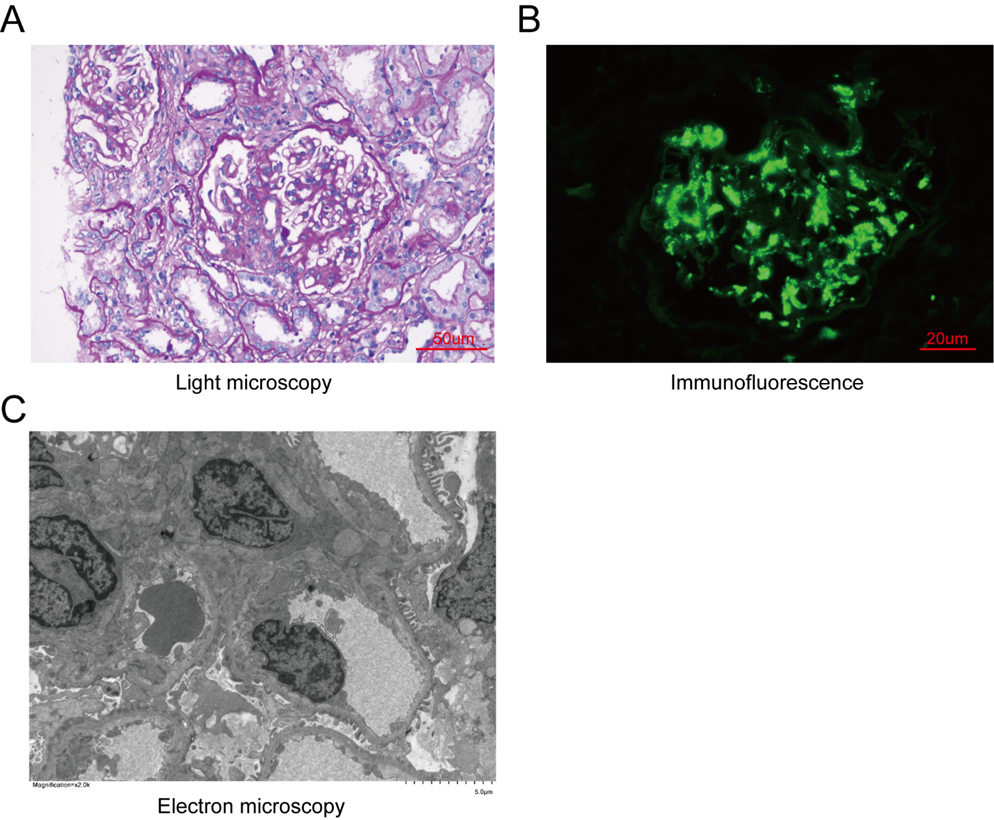


**Figure S3. Representative histopathological findings of IgA nephropathy** (A) Light microscopy: mesangial proliferation (periodic acid–Schiff, ×200); (B) Immunofluorescence: mesangial staining of IgA (×400); (C) Electron microscopy: mesangial deposits along with mesangial matrix expansion and cellular proliferation (×2000).


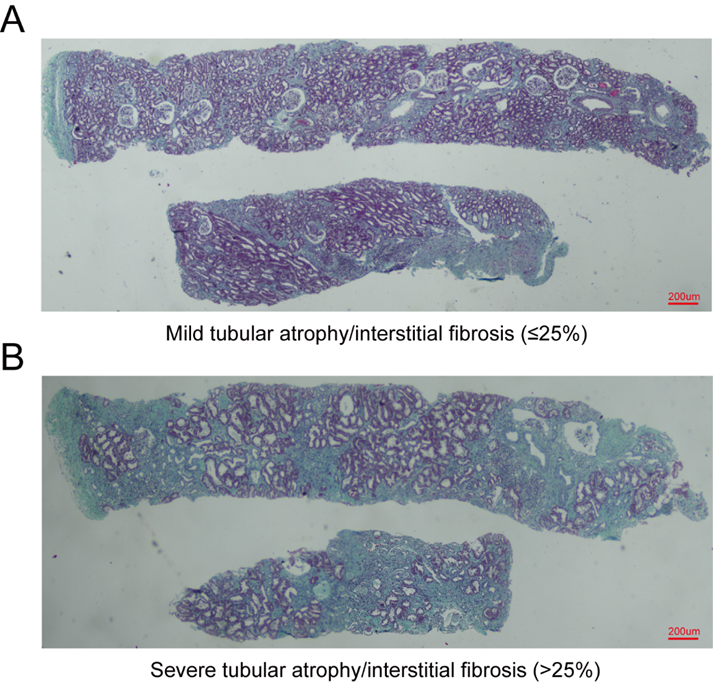


**Figure S4. Representative histopathological images of mild and severe tubular atrophy/interstitial fibrosis in IgA nephropathy** (A) Mild tubular atrophy/interstitial fibrosis (≤25%) (Masson, ×40); (B) Severe tubular atrophy/interstitial fibrosis (>25%) (Masson, ×40).


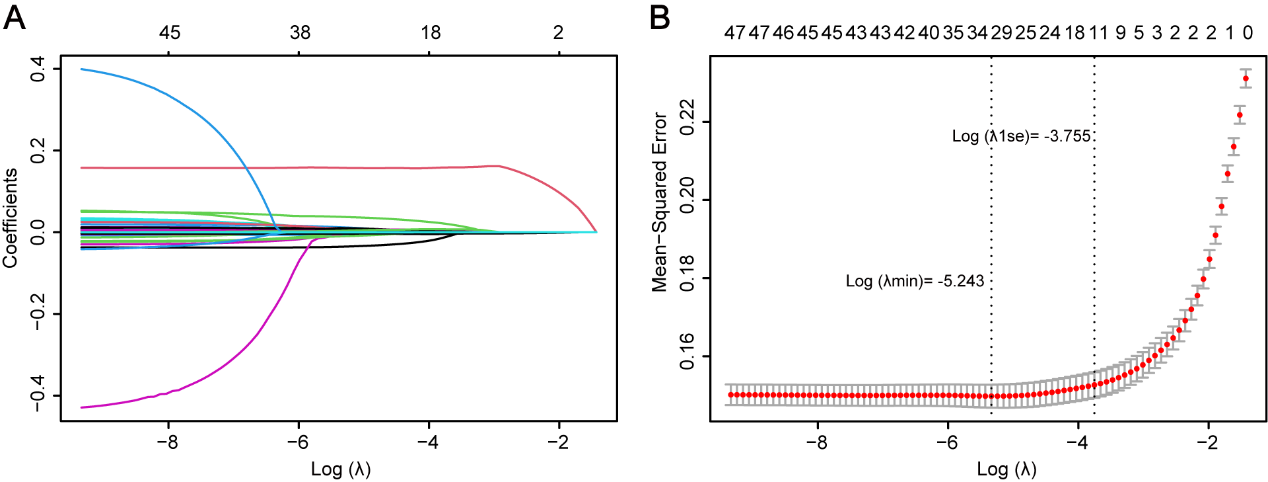


**Figure S5. Variable selection process for tubular atrophy/interstitial fibrosis using LASSO regression.** (A) Coefficient path plot; (B) Cross-validation curve.


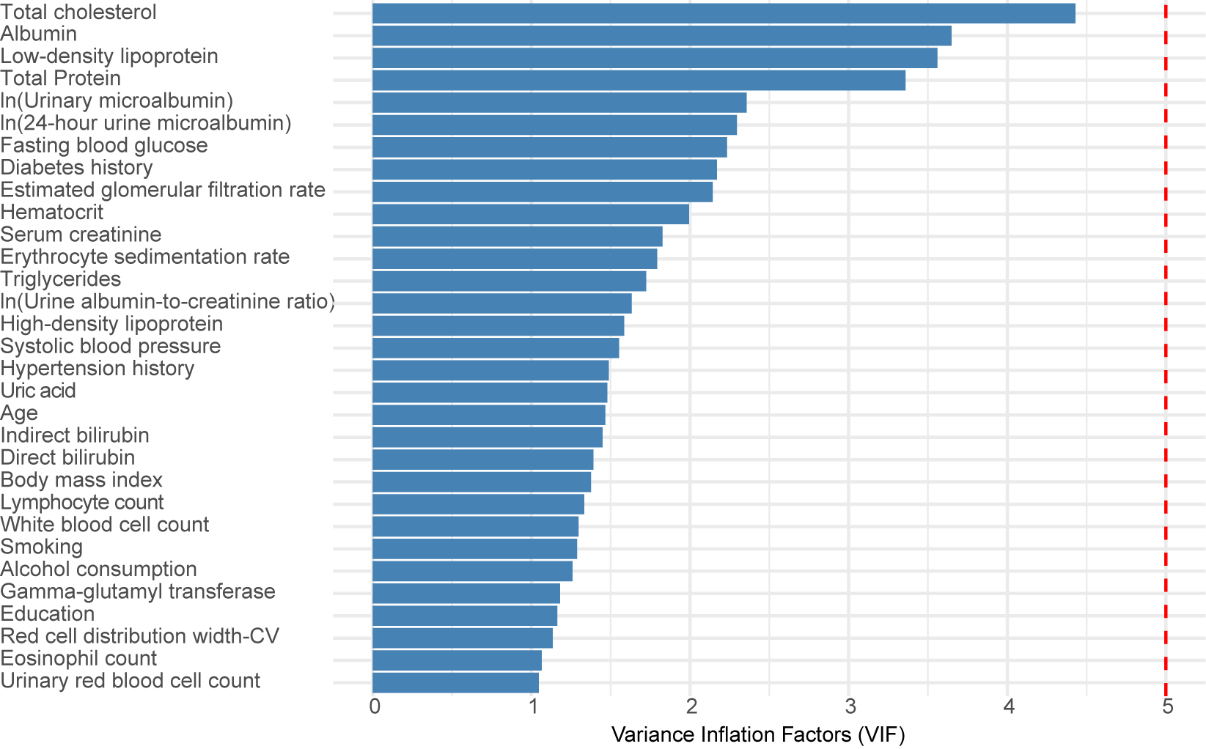


**Figure S6. Variance inflation factor (VIF) ranking of 31 candidate predictors for severe tubular atrophy/interstitial fibrosis.**
